# Action representation in the mouse parieto-frontal network

**DOI:** 10.1038/s41598-020-62089-6

**Published:** 2020-03-27

**Authors:** Tuce Tombaz, Benjamin A. Dunn, Karoline Hovde, Ryan John Cubero, Bartul Mimica, Pranav Mamidanna, Yasser Roudi, Jonathan R. Whitlock

**Affiliations:** 1 Kavli Institute for Systems Neuroscience & Centre for Neural Circuits, Norwegian University of Science and Technology, Trondheim, Norway; 2 Department of Mathematical Sciences, Norwegian University of Science and Technology, Trondheim, Norway; 3 Department of Health Science and Technology, Center for Sensory-Motor Interaction, Aalborg University, Aalborg, Denmark; 4 Present Address: Institute of Science and Technology (IST) Austria, Klosterneuburg, Austria

**Keywords:** Sensorimotor processing, Social neuroscience

## Abstract

The posterior parietal cortex (PPC) and frontal motor areas comprise a cortical network supporting goal-directed behaviour, with functions including sensorimotor transformations and decision making. In primates, this network links performed and observed actions via mirror neurons, which fire both when individuals perform an action and when they observe the same action performed by a conspecific. Mirror neurons are believed to be important for social learning, but it is not known whether mirror-like neurons occur in similar networks in other social species, such as rodents, or if they can be measured in such models using paradigms where observers passively view a demonstrator. Therefore, we imaged Ca^2+^ responses in PPC and secondary motor cortex (M2) while mice performed and observed pellet-reaching and wheel-running tasks, and found that cell populations in both areas robustly encoded several naturalistic behaviours. However, neural responses to the same set of observed actions were absent, although we verified that observer mice were attentive to performers and that PPC neurons responded reliably to visual cues. Statistical modelling also indicated that executed actions outperformed observed actions in predicting neural responses. These results raise the possibility that sensorimotor action recognition in rodents could take place outside of the parieto-frontal circuit, and underscore that detecting socially-driven neural coding depends critically on the species and behavioural paradigm used.

## Introduction

A key function of any motor system is the rapid and flexible production of actions in response to external stimuli, including the behaviour of other individuals. Having robust representations of performed and observed behaviours has been hypothesized to add survival value in a number of species since it could facilitate optimal action selection, gaining access to food sources or avoiding predators^1^. However, which neural circuits integrate performed and observed actions, and how, are not well understood. In different species of primates and songbirds, a striking manifestation of such interactions has been described in the form of mirror neurons. Mirror neurons, first characterized in pre-motor cortex^2,3^ then PPC^4^ in monkeys, and later reported in humans^5^ and birds^6^, respond reliably both when an individual performs a specific action and when they observe the same action performed by a conspecific. Based on these properties they have been postulated to enable specific social functions ranging from selecting appropriate actions in response to observed behaviours^2^ to understanding the intentions and imitating the actions of others^7,8^. After years of investigation, however, it is still debated whether mirror cells are at the basis of action understanding or if their physiological properties can be better explained by simple, temporally contingent sensory-motor associations^9^. Finding mechanistic resolutions to these questions would benefit tremendously if it were possible to access cellular networks underlying social learning in a genetically tractable animal model.

We therefore sought to establish whether mirror-like neurons occur in frontal motor areas or PPC in mice. Like other rodents, mice can socially acquire both sensorimotor and fear-based behaviours^10–15^, and they have recently proven effective models for studying the neurobiology of empathetic social learning^16–20^. Emerging evidence also suggests that PPC and M2 in rodents, as with primates, comprise a cortical network supporting several aspects of goal-directed behaviour, including decision making^21,22^, sensorimotor transformations^23,24^, and movement planning^25,26^. Rodent models also bring methodological advantages including large-scale neural recordings in unrestrained subjects, which enable the analysis of neural ensemble dynamics during any number of self-initiated, naturalistic actions. In turn, it is possible to uncover intrinsic features of neural population activity driven by behaviour, such as state-space structure, independently of experimenter bias^27^.

Here, we used miniaturized, head-mounted fluorescent microscopes^28^ to image the activity of hundreds of individual neurons at a time while mice performed or observed pellet-reaching and wheel-running tasks. We confirmed that observer animals were attentive and showed reliable neural responses to visual stimuli in the task. By applying dimensionality reduction to recordings from the behavioural experiments^29^, we found clear differences in the structure of ensemble responses during performed and observed behaviours. This motivated the subsequent quantification of single-cell selectivity to specific behaviours using shuffling analyses as well as statistical modelling with a generalized linear model (GLM). All tests indicated that PPC and M2 were driven strongly by performed behaviours, similar to what has been shown in more stereotypical tasks^27^, but extended here to freely behaving animals. The neural coding of observed behaviour, on the other hand, was below chance levels in both brain areas, even in neurons with strong performance correlates. These results indicate that the representation of the observed actions we tested occurs outside the parieto-frontal circuit in mice, or that such representations in rodents require additional sensory input beyond vision.

## Results

**Figure 1:**
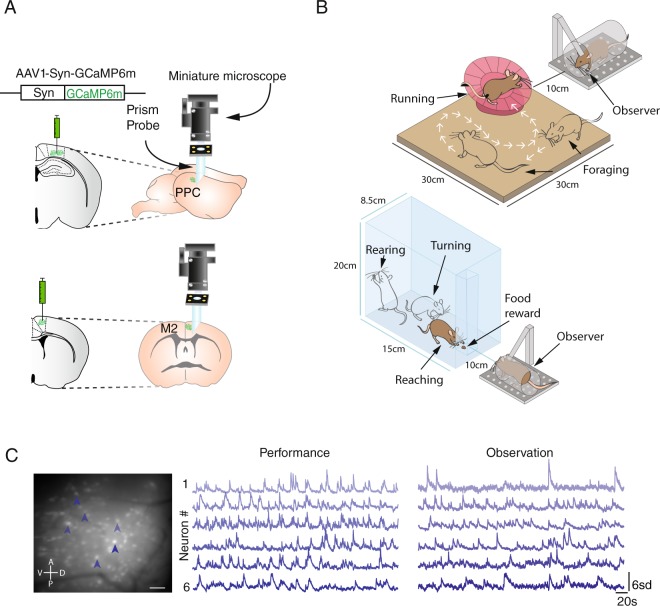
Experimental paradigm for imaging neural populations in PPC and M2 in freely behaving mice. (**A**) PPC and M2 were transfected virally to express GCaMP6m (*Left*), and miniature prism probes were implanted to image tangentially across cortical layers (*Right*) during different behavioural tasks. (**B**) In the experiments, mice alternated between performing and observing a conspecific in a pellet-reaching task (*Bottom*) and wheel-running task in an open arena (*Top*). Dynamic calcium fluctuations were monitored in each mouse during four 10-min recording sessions, two of which were during performance and two during observation of each task. (**C**) (*Left*) Average of 500 images of the entire FOV after image pre-processing. Scale bar, 100 µm. Shaded arrows indicate 6 cells whose calcium traces are shown (*Middle*) during performance and (*Right*) observation of the pellet-reaching task.

To determine whether neurons in PPC and M2 reliably responded to the performance and observation of the same set of behaviours, we used one-photon epifluorescence microscopy to image the activity of neuronal ensembles expressing the genetically encoded calcium indicator GCaMP6m (AAV1.Syn.GCaMP6m.WPRE.SV40) via AAV-mediated transfection (921 neurons in PPC in 4 mice; 852 neurons in M2 in 4 mice; Fig. S2, Table S1). Cellular responses were monitored through a chronically implanted gradient refractive index lens attached to a prism (Fig. 1A,C). All animals were trained to perform the pellet-reaching task in an 8.5 × 15 × 20 cm box (Fig. 1B), in which they were taught to reach through a 1 cm diameter hole to grasp food pellets (Fig. 1B). They were trained to asymptotic performance levels prior to experimental recordings (maximum of 10 days; Methods) and, concurrently, were habituated to head-fixation and to observe a sibling perform the same task. In the experiments, each animal’s cortical activity was imaged during four sessions, with performance (P) and observation (O) conditions interleaved (following a P1-O1-P2-O2 scheme). In parallel, we recorded from each mouse while they behaved freely in a wall-less open arena (30 × 30 cm) with a running wheel, and while they observed a sibling doing the same (Fig. 1B). The calcium imaging data were paired with high-resolution behavioural recordings made during both performance and observation sessions.

Several precautions were taken in the design of the observation condition to minimize potential experimental confounds. This included head-fixing the observers, which reduced contamination related to the observers’ bodily movements in observation-related neural signals and served to train the observers’ viewing angle on the behavioural tasks (Fig. 1B). The observer mice were habituated to this procedure gradually so as to minimize stress-related motion during recordings (Methods). Furthermore, we controlled for the influence of spontaneous movements on the neural data either by discarding neurons tuned to the observers’ body movement in single-cell analyses or by including movement as a covariate in our statistical models. We also avoided training the observers to report when they had observed a behaviour since such movements would interfere with observation-related neural responses. Instead, we monitored the pupil diameter and bodily movement of the observers as proxies for attention and arousal^30,31^, and confirmed with statistical modelling that these features changed predictably as a function of the performers’ actions (Fig. S1A,B). To make sure that the limitations in mouse vision did not compromise our results, we positioned the observing mice no more than 10 cm away from the apparatus, a distance comparable to those reported in studies demonstrating observational learning in mice^14,15^. We further confirmed that the observers could see moving objects at these distances by placing and removing food pellets in front of them with forceps, which produced sufficiently strong neural responses to accurately decode whether forceps were present or not during the recording (Fig. S1C–F).

**Figure 2:**
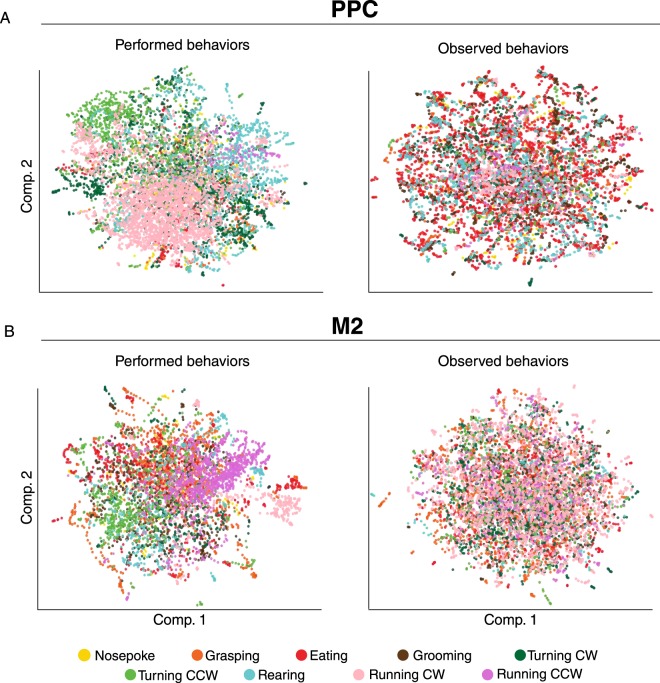
UMAP projections of population activity in both PPC and M2 reveal structural segregation for performed but not observed behaviours. (**A**) PPC ensemble activity of one mouse separated in the reduced dimensional space during specific performed behaviours, including wheel-running (beige dots), counter-clockwise turning (light green) and rearing (blue). By contrast, the distribution of points during observed behaviours (*Right*) was spread homogeneously in UMAP space. Each dot corresponds to the activity state of the entire population of recorded neurons at a given time point; colour-coding for each behaviour is shown at bottom. (**B**) Recordings from the same M2 mouse were similar to *A*, showing a stronger tendency to cluster during performed than observed behaviours. Example mice having the highest Dunn index ratio between performance and observation sessions are shown.

Having imaged large ensembles of neurons in PPC and M2 from performing and observing mice, as a prelude to our analysis, we visualized how performance and observation conditions affected the population activity. To this end, we applied the uniform manifold approximation and projection (UMAP) method on downsampled population activity vectors (Methods)^29^. As shown in Fig. 2A,B, this revealed structural discrepancies in the dimensionally-reduced activity space between performance and observation sessions, with population activity states being closer to each other for time points belonging to the same behaviour during performance, but not observation conditions (Movie S1). We measured the degree to which time points labelled by the same behaviours were clustered using the Dunn Index (Methods), which produced clustering indices between 2.4 and 10.9 times higher during performance than observation sessions across animals (3 mice in PPC, 1 mouse in M2). This suggested that there were clear signatures of the representation of performed behaviour but not observed behaviour in PPC and M2 activity. Due to the dependence of the quantitative aspects of the UMAP results on several initial parameters, such as the dimension of the projective space, a more careful quantification of these effects required going beyond this visualization, which is what we report in the rest of the paper.

**Figure 3:**
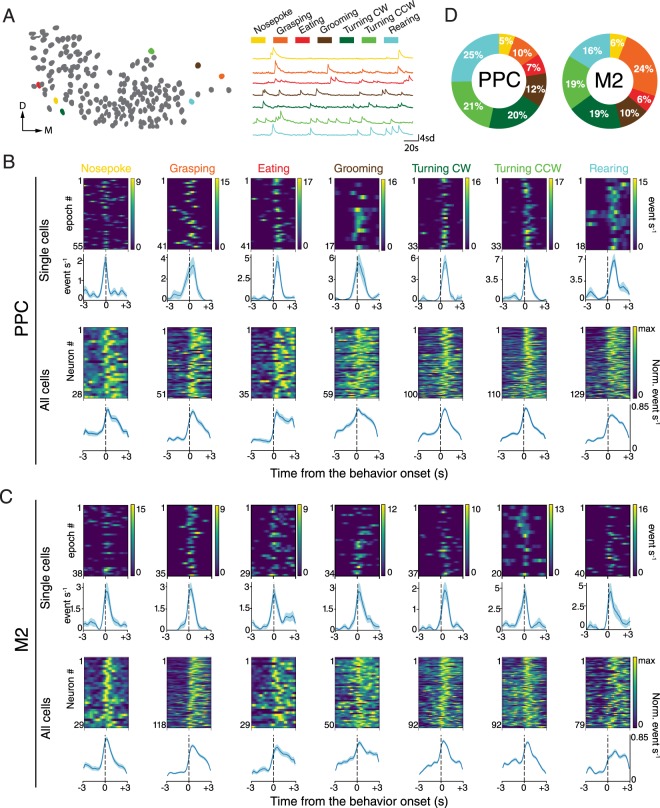
Cell populations in PPC and M2 robustly encode actions performed in the pellet-reaching task. (**A**) Representative neural map (*Left*) and Ca^2+^ transients of 7 PPC neurons (*Right*) tuned to each of the behaviours in the pellet-reaching task; colour coding for each behaviour is shown above. (**B**) (*Top*) Temporal profiles of behaviourally evoked responses of single cells for each behaviour are shown as heat maps; immediately beneath are behaviourally aligned average activity rates for each cell over the entire session. (*Bottom*) Normalized responses for all behaviourally tuned PPC neurons from all animals aligned to behaviour onset; population means are shown in the row underneath. Colour bars indicate max event/s; blue shaded regions around averaged rates denote ± SEM. (**C**) Same as *B*, for single cells (*Top row*) and cell populations (*Bottom row*) imaged in M2. (**D**) Colour-coded pie charts indicating the proportion of stably tuned neurons for each behaviour; a total of 1645 cells were imaged in 4 mice in PPC; 1068 cells were imaged in 4 mice in M2.

To determine if the UMAP results reflected behavioural selectivity at the single-cell level, we quantified the tuning of individual neurons to different actions the animals engaged in while performing the tasks. We labelled the onset and offset of discrete, recurring behaviours, including turning left or right, nose poking, grasping to eat, eating, rearing or grooming (Fig. 3A; Movie S2; Methods). A cell was considered stably tuned to a behaviour if its in-behaviour event rate exceeded 95% of the shuffled in-behaviour rates in two consecutive performing sessions (Methods). Approximately half the neurons in both PPC (430 of 921 cells; 46.6% in 4 mice) and M2 (439 of 852 cells, 51.5%, 4 mice) were reliably driven by performed behaviours (Fig. 3A,C; Table S1). While the majority of neurons were uniquely tuned to individual behaviours, subsets of cells were selective for multiple actions, and in all cases, tuning was invariant to the duration of the behaviour (Fig. S3). In the open field task, 67 of 724 PPC cells (9.3%; 3 mice) were stably tuned to clockwise (CW) and counter-clockwise (CCW) wheel-running, while 21 out of 216 neurons (9.7%, running CCW only; 1 mouse) were stably tuned in M2. The proportion of cells representing each behaviour varied between animals, with larger groups of cells tuned to turning in both PPC and M2, and a larger proportion of cells tuned to grasping in M2 than PPC (Figs. 3B–D, S3). PPC neurons tended to favour the forelimb contralateral to the recoding sites, which were always in the right hemisphere (24% and 11% of tuned cells preferred left-handed grasping in two animals *vs*. 6% and 7% for right-handed grasping in the other two), whereas limb preference in M2 was less specific (21% and 41% for left-handed grasping, and 37% and 12% for the right) (see also^32^).

The heterogeneity of tuning properties, and the tuning of some cells to multiple behaviours, raised the question as to whether cells with similar coding clustered anatomically, as suggested by prior work in parietal and motor areas in different mammalian species^33–35^. Ensemble imaging allowed us to assess the spatial micro-organization of behaviourally responsive neurons according to their tuning preference in each brain region of each animal. However, an analysis of the quality of clustering by behavioural tuning (Dunn Index; Methods) showed no clear tendency of grouping between cells with similar properties, nor any clear mapping based on cortical depth or location in the imaging field of view (Figs. S4, S5).

**Figure 4:**
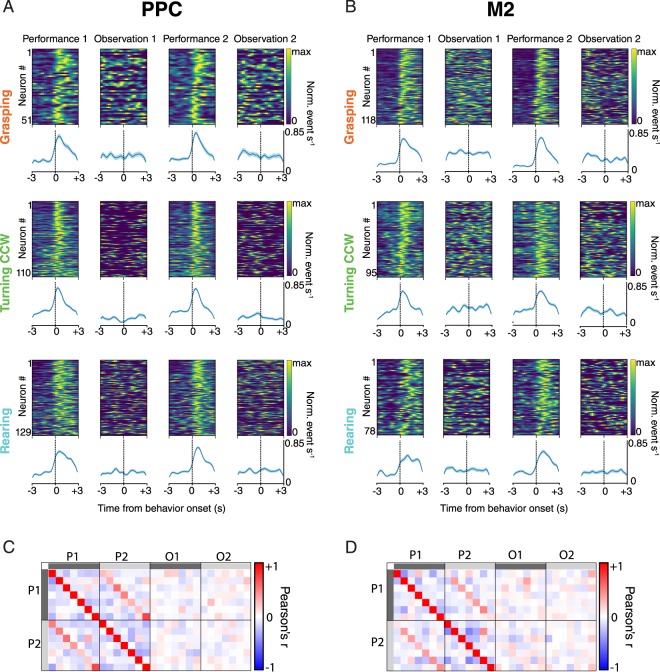
Neural ensembles in PPC and M2 stably represent performed, but not observed actions. (**A**) (*Above*) Session-averaged Ca^2+^ responses of individual cells aligned to the onset of specific actions in the pellet-reaching task and ranked by z-scored firing rate during the first performance session (P1). (*Below*) Population average (± SEM) of responses of all cells for each behaviour. Virtually none of the cells with stable correlates across the two performance sessions responded when the same actions were observed (Observation sessions 1 and 2), yielding a flat activity rate in the ensemble average. (**B**) Same as *A*, but for cells recorded in M2. (**C**) Correlation matrices, with each square corresponding to a particular behaviour, show the sustained specificity of behavioural tuning in PPC across performance sessions (P1 and P2). The conserved correlation structure is reflected by the red diagonal in P1 vs. P2, which is notably absent across performance and observation conditions. (**D**) Same as *C*, for recordings in M2.

Since PPC and M2 showed robust tuning to a variety of performed behaviours, we next assessed whether they responded during observation of the same actions. We compared trial-averaged responses to specific behaviours across all four recording sessions: P1, O1, P2 and O2 (Fig. 4A,B upper panels). However, in both brain areas and across mice, we saw negligible neural tuning to observed actions, irrespective of whether the cells stably encoded performed actions (Fig. 4A,B lower panels, Fig. S6). Specifically, 15 of 921 neurons (1.6%) in PPC and 13 out of 852 neurons (1.5%) in M2 exhibited stable observational correlates for the pellet-reaching task, even though the total amount of time the animals spent observing behaviours was comparable to the time spent performing them (Table S1). The few cells with observation correlates did not show apparent anatomical localization, either within or across animals. To test whether the proportion of such cells exceeded chance levels, we paired neural activity with behaviour labels from the wrong sessions and computed false-positive rates for all sessions and all animals (Methods). This approach identified 27/921 (2.9%) PPC cells and 32/852 (3.8%) M2 cells as stably tuned to mismatched observed behaviours, demonstrating that the number of stable observational correlates was below chance (PPC: U = 286.5, p > 0.05, M2: U = 228, p > 0.05; Mann-Whitney U test). Similarly, in the open field task, only three out of 724 neurons (0.4%) and one out of 216 neurons (0.5%) had reliable observational tuning to running behaviours in PPC and M2, respectively. Fewer than 1% of cells had stable, matched correlates for performed and observed actions in the pellet-reaching task in either area, which again was below mismatched data rates (U = 364, p > 0.05 for PPC; U = 287.5, p > 0.05 for M2). Moreover, no cells showed matched tuning for wheel-running behaviour in the open field task (Table S1).

To investigate whether the lack of neural responsiveness to observed actions stemmed from fluctuations in arousal state, we measured variations in pupil diameter, a proxy for arousal and attention^30^, in a subset of mice. Since prior work established that contraction of the pupil is associated with reduced attentiveness and neural responsiveness^31^, we restricted our analyses of observation sessions to exclude epochs when the pupil diameter was smallest (Fig. S7; n = 3 animals). Consistent with our prior findings, however, this did not affect the number of cells showing stable tuning (9 of 621 cells (1.5%) with all timepoints included, 8 cells (1.3%) when excluding pupil contraction), indicating the lack of effect did not relate to low arousal of the observers.

To further assess whether activity patterns during performance sessions related to observation, we calculated how well cellular activity could be predicted from one task condition to another. When cells were selected based on their behavioural tuning in the first performing session, and their z-scored firing rates were correlated to those in the second performing session, we saw in every case that the responses of cells correlated positively (Pearson’s correlations for same-behaviour comparisons ranged from 0.22 to 0.75 for PPC and 0.08 to 0.48 for M2; Fig. 4C). Likewise, selecting cells based on their tuning in the second session and correlating those rates back to the first yielded similar results (r-values ranged from 0.20 to 0.71 in PPC and 0.1 to 0.44 for M2; Fig. 4C). By comparison, the correlations between performance and observation sessions centred around zero in all cases (r-values ranged from −0.09 to 0.12 for PPC and −0.24 to 0.22 for M2; Fig. 4C).

**Figure 5:**
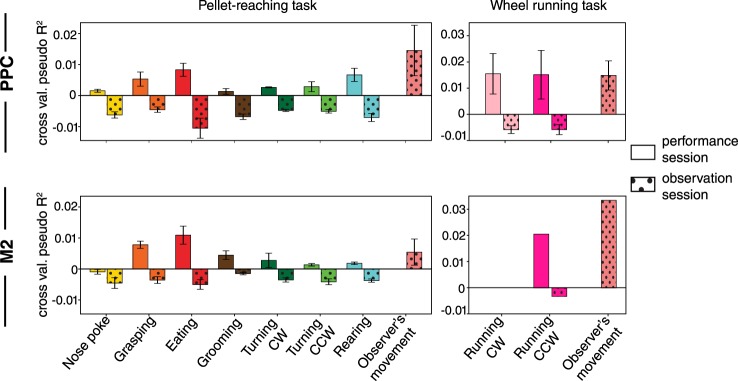
Bar plots show the cross-validated pseudo-R^2^ for a single behaviour Bernoulli generalized linear model of calcium events from neural populations in PPC (*Top panels*) and M2 (*Bottom panels*) of mice during the pellet-reaching task (*Left panels*) and wheel-running task (*Right panels*). Hand-labelled behaviours from performance sessions are shown as empty boxes while behaviours from observation sessions as hatched boxes. Bars represent the mean ± SEM over animal subjects (pellet-reaching task: 4 mice for PPC and 4 animals for M2; wheel-running task: 3 mice for PPC and 1 mouse for M2).

Lastly, we wished to determine the extent to which each of the behaviours explained the activity rates of the cells during performance and observation conditions, for which we used a generalized linear model (GLM) framework (Methods). The model was designed to incorporate all labelled behaviours as predictors of each neuron’s time-varying activity. To quantify how well the behavioural variables accounted for the activity of the neurons, we computed the cross-validated pseudo-*R*^2^ by taking the difference between the log-likelihoods of the null-model and of the single variable models normalized to the former model^36^ (Methods). For each of the behaviours considered, and in both pellet-reaching and open field tasks, we found that neural responses in PPC and M2 were better predicted by performed behaviours compared to a model with only the constant term (i.e. the mean firing rate; Fig. 5). We also noted that the proportions of neurons that were stably tuned to task-dependent behaviours such as grasping (10% in PPC and 24% in M2) and eating (7% in PPC and 6% in M2) fared better than those with task-independent behaviours, such as grooming or rearing. Predictions based on observed behaviours, on the other hand, were in all cases worse than the null-model (Fig. 5), which was contrasted strongly by the significant improvement in model performance for the observers’ own movements.

## Discussion

The results of our study demonstrate that PPC and M2 were reliably modulated by the execution of various natural behaviours in both pellet-reaching and wheel-running tasks, which was juxtaposed sharply by the low number of neurons responding to observed behaviours, which neither exceeded chance levels nor aided in predicting neural activity. Our analysis was inspired by exploration of the dimensionally- reduced network state dynamics across task conditions, which revealed that population activity in both brain areas was more structured during performance than observation of behaviours. We note that the behavioural clusters in the dimensionality-reduced manifold of performance sessions were not fully separated, which could suggest that the population vectors do not lie completely on a two-dimensional non-linear manifold, that other tunable parameters of UMAP were not ideally chosen, or that variables which we did not measure, such as posture or decision-making, bind separate behaviours more closely together. In contrast, action observation did not elicit any appreciable structure in population activity. This led us to perform a GLM analysis which confirmed that action observation does not predict neural activity. In fact, the bodily movement of the observers was the most influential factor in the statistical model, which was consistent with results from the performance sessions, and could have been part of a larger wave of neural activation throughout the brain, as described in recent work in head-fixed animals^37,38^.

The fact that the animals were freely moving when performing the tasks allowed us to measure how cells responded to a variety of actions, revealing new features of behavioural coding in both PPC and M2. First, approximately 15% of cells in both areas stably represented more than one behaviour (Fig. S3), and cells coding for different behaviours were intermingled anatomically. This indicates that cell ensembles in PPC or M2 are apt to participate in more than one behavioural representation, though any overarching organization of tuning based on somatotopy^33^, posture^39^ or ethological organization^34,40^ was not apparent at the microscales at which we were imaging. The exact proportion of represented behaviours varied per animal, though both PPC and M2 showed strong behavioural tuning, with PPC showing more prevalent correlates for turning and rearing, and M2 with stronger representation of grasping with the forelimb. In both areas, however, eating was the best predictor of population calcium events in the GLM (Fig. 5), despite that it was coded by comparatively few neurons. Since this predictability could not be attributed to the over-expression of eating epochs relative to other behaviours (Fig. S3), it could reflect the salience of the consumptive behaviour. It could also imply a population coding strategy where increased single-neuron selectivity compensates for the small population size or, conversely, that a small population size is all that is used because the neurons are strongly tuned^41^. On the whole, such heterogeneous response selectivity across behavioural categories is consistent with previous work on multisensory coding and decision making in the rodent PPC^42,43^, and the absence of spatial clustering for similarly tuned neurons is consistent with the dispersed anatomical organization of orientation tuning in primary visual cortex^44^, olfactory coding in the piriform cortex^45^, and choice-selectivity in PPC^46^.

As for mirror neurons, they have been best characterized across primate species in pre-motor cortex and PPC which, together, comprise the parieto-frontal network^2,4,47,48^. This network supports several functions required for goal-directed behaviour including sensorimotor transformations, action planning and decision making^49^. Although it was long thought that rodent brains lacked the prerequisite complexity to subserve higher cognitive functions, a growing body of work shows that both rats and mice exhibit accomplished performance in sensory-motor tasks such as virtual navigation^46^ and evidence-based decision making^21,50^, and they show stimulus history effects^51–53^. Though PPC and M2 are considerably less elaborate in rodents than primates, both in terms of relative size and the number of subfields^54^, there are several anatomical features common to both species which could support action recognition, including strong input from higher visual areas^55,56^ and topographically organized reciprocal connections linking PPC with frontal motor cortices^57–61^. Given the anatomical and functional similarities, we reasoned that neurons in the rodent parieto-frontal circuit might exhibit mirror-like responses to the observation and execution of the same actions and were surprised by the effective absence of observational tuning in both areas.

To our understanding, there are at least three possibilities why this could be the case. One is that the pellet-reaching task encapsulated actions that were not affectively salient for rodents. Although grasping and eating behaviours, which elicit mirror neuron activation in primates^2^, and wheel-running were strongly represented in the brains of the performers in our study, we cannot conclusively rule out that the task structure failed to evoke sufficient arousal in the observers. In that sense, our task and findings contrast with aversive social learning paradigms^19^, where, for example, mirror-like responses have been shown for pain in the anterior cingulate of rats^20^. Such paradigms evoke autonomic responses and basic survival instincts, which may be instrumental in attracting sufficient attention in a rodent observer. A second, related possibility is that rodents may need to interact physically for neural responses in one animal to reflect the actions of a cohort, such as during behavioural dominance paradigms^62^. We note, however, that disentangling neural signals for performed and observed actions in such a task would necessitate continuous monitoring of posture and muscle tone in both subjects, which is a technical obstacle we sought to overcome by head-fixing the observers. Head-fixation, however, enforced a physical distance between the animal pairs, which in turn limited their ability to sensorially sample each other during the tasks. The requirement for behavioural control may thus prove a major challenge for demonstrating sensorimotor mirroring in rodents, since they rely heavily on sensory systems other than vision, like olfaction and audition, which are gated by social proximity^63^. A final possibility for our findings is that observed actions are encoded in areas upstream or outside of the posterior parietal and motor areas we imaged. For example, extrastriate areas AL and RL receive the same, if not more, input from V1 as the more medial regions we imaged in PPC, and they also project to frontal motor cortices. Additionally, areas shown to respond in other social paradigms, like the mPFC^62^ or the anterior cingulate cortex^19,20^, could be potential targets for sensorimotor-based experiments in the future.

The overall goal of the present experiments was to determine if performed and observed actions are represented in mice, in cortical areas corresponding to those where mirror neurons were discovered and studied most thoroughly in primates. Although we did not find mirroring properties in PPC or M2, the absence of evidence does not equate to evidence of absence. Our experiments utilized one strain of laboratory mice and there were both technical and biological constraints that preclude strong conclusions, for example, about the phylogenetic development of the mirror system. Future studies will need to account for species-specific constraints in their experimental design, because the likelihood of finding mirror neurons will vary depending on the animals used, their natural ecology and the specific neural systems investigated.

## Materials and Methods

### Subjects and virus injection

The experiments were performed in accordance with the Norwegian Animal Welfare Act and the European Convention for the Protection of Vertebrate Animals used for Experimental and Other Scientific Purposes. All experiments were approved by the Norwegian Food Safety Authority (Mattilsynet; protocol IDs 6833 and 16356). Experimental mice were 3 to 7-month-old wild type C56BL/6 females (6 from Taconic Bioscience, 2 from The Jackson Laboratory), individually housed on a 12 hr inverted light/dark cycle with ad libitum access to food and water. Surgeries were performed under sterilized conditions and body temperature was maintained at 37 °C with a heating pad. Anaesthesia was induced using isoflurane mixed with oxygen (5% for induction, 1–1.5% for maintenance) on a stereotactic frame (David Kopf Instruments). Prior to surgery, mice were injected with analgesics subcutaneously (Metacam 1 mg/kg, Temgesic 0.1 mg/kg weight) and with a local anaesthetic (Marcain 0.5 mg/ml) under the skin surface above the skull before making an incision. Following the initial induction and drug administration, the dorsal surface of the head was shaved and ophthalmic ointment was applied to the eyes. The incision area was scrubbed with cotton swabs dipped in 70% Ethanol followed by betadine (2 x each), and a small incision was made along the midline. All measurements were made relative to bregma for virus and prism probe implant surgeries. A craniotomy (1.2 × 1.2 mm) was made and each animal was injected with 300 nl of AAV1.Syn.GCaMP6m.WPRE.SV40 (University of Pennsylvania Vector Core; item # AV-1-PV2823) at multiple locations in the right hemisphere of the posterior parietal cortex (AP: −1.95, ML: 1.5, DV: 0.35 and 0.7; AP: −1.95, ML: 1.9, DV: 0.35 and 0.7 mm relative to bregma) or secondary motor cortex (AP: +0.5, ML: 0.5, DV: 0.5; AP: +0.2, ML: 0.5, DV: 0.5 mm relative to bregma) using a Nanoject II Injector (WPI, USA), delivering virus at a rate of 35 nl per min with a controller (Micro4; WPI). The glass injection pipette was left in place for 10 min post-injection, after which it was slowly withdrawn. Following the viral injections, the craniotomy was filled with Kwik-Sil silicone elastomer (WPI) and the incision was closed with nylon sutures. After surgery, mice were kept in a heated chamber until they regained consciousness and began moving.

### Prism probe implantation

One week post virus-injection, a 1 mm diameter gradient refractive index lens (GRIN) attached to a prism (Inscopix) was lowered stereotaxically into the craniotomy at a rate of 10 µm/s while the tissue was treated constantly with saline to minimize desiccation. The prism lens was positioned 1.2–1.3 mm deep and 0.15–0.2 mm away from the injection site. Lens implants were secured to the skull with a thin layer of Kwik-Sil silicone elastomer, followed by a thick layer of adhesive cement (super-bond C&B, Sun Medical). The lens cuff was filled with Kwik-Cast (WPI) for protection during a 1–2 week interval to allow for viral expression. A custom-made head bar was cemented to the skull with dental acrylic for head fixation in behavioural experiments.

Once viral expression was confirmed, mice underwent anaesthesia to secure a baseplate (Inscopix), which was cemented on the prism probe to support the connection of the miniaturized microscope during *in vivo* imaging under freely moving conditions. During the procedure, a baseplate was attached to the miniature epifluorescence microscope (nVista HD, Inscopix) and stereotaxically positioned to a desired focal plane with the help of visible landmarks (GCaMP6m-expressing neurons and blood vessels) using 20–30% LED power, a frame rate of 5 Hz and digital gain of 4. Once the focal plane was identified, the microscope and baseplate were raised by ~50 µm to compensate for shrinkage of the adhesive cement and were subsequently fixed in place using the same compound, followed by a thin layer of dental acrylic mixed with black carbon spherical powder (Sigma Aldrich) to minimize the light interference of the imaging field. The baseplate was covered with a protective cap (Inscopix), and imaging began within 1–2 days.

### Behavioural training and recording

Animal training. Pairs of sibling animals were used in all experiments and were housed together for one week prior to the start of training. During this period, each animal was habituated to the experimenter and handled extensively on a daily basis. Subsequently, mice were housed individually and food-restricted to maintain 90% initial body weight throughout the training period. They were trained daily for 7–10 days in a modified version of the pellet-reaching task^64^. The chamber used for the task was built from clear plexiglass (3 mm thick, 20 × 8.5 × 15 cm) with a rectangular cylinder attached externally through which food pellets were delivered (Fig. 1). After one day of habituation to the box with no pellets, animals underwent 2 stages of task acquisition; shaping and training. During shaping (2 days, 2 sessions per day), mice were presented with multiple chocolate pellets (20 mg per pellet, TestDiet) in the reaching compartment to reinforce reaching behaviour. During the subsequent training period (5 days, twice per day), a single pellet was placed in the reaching compartment and the animals’ performance was monitored during 15 min sessions. In this task, each mouse learned spontaneously to turn in a circle in place to elicit pellet delivery (leading to a turn-grasp-eat motif), though this was not explicitly shaped by reinforcement. Trials in which animals retrieved the food pellet with their tongue were excluded from the analysis. Experiments began once mice exceeded 40 successful trials in at least 2 consecutive sessions.

Following head bar placement, the same cohort of animals was gradually habituated to head-fixation over an 8–10 day period. First, they were allowed to move freely in and out of a 4.5 cm diameter acrylic tube and were subsequently head-fixed with their body in the tube for 15 min. Over 7 days this was increased to 45 min until body movement was minimal. Finally, animals were habituated to head fixation while another conspecific performed the pellet-reaching task in front of them. This process typically required ~10 days.

After the pellet-reaching task, mice were placed in a wall-less, open, squared arena (30 × 30 cm) with a running wheel, and allowed to behave freely during 20 min sessions. The animals were pre-trained until they exhibited full coverage of the arena, and the same cohort was head-fixed in the tube and, alternately, performed or observed siblings perform the open field task.

Behavioural recording setup. The animals’ behaviour was recorded with 5 high-resolution, near-infrared (NIR) cameras (4MP, 100fps, 850 nm; Simi Reality Motion Systems GmbH, Germany): one capturing both the performer and observer, one solely on the observer and three exclusively on the performer. The cameras were angled to minimize redundancy of view, and infrared illumination was aided by 8–10 additional NIR LED lamps (850 nm, 48 LEDs each; Banggood). All experiments were performed in dim visible light with the experimenter hidden from the view of the animal.

### Pupil measurements

To control for changes in arousal state and neural responsiveness during observation sessions^31^, variations in pupil size were measured for 3 mice using close-up video from the camera positioned specifically on the observer, with additional NIR (850 nm) illumination of the left eye (Fig. S7). ImageJ software (NIH, version 1.52e) was used to trace a region of interest (ROI) at the lateral edge where the pupil, which was black, met the lighter-coloured sclera, which changed dynamically when the pupil dilated or contracted (as in ref. ^65^). The mean pixel intensity of the ROI was registered as a negative number that was closest to zero (i.e. largest) when the pupil was dilated maximally and was most negative when the pupil was contracted (Fig. S7). For each mouse, a binary threshold was determined that captured periods when the pupil was contracting to the smallest size; this was used to flank epochs when the pupil was most contracted, typically when animals were quiescent and motionless.

### Behavioural labelling

Videos were decompressed and downsampled by a factor of 5 (except for one animal which had a 25fps image acquisition rate) to reduce file size and match calcium imaging sampling frequency. The videos of several behavioural sessions were reviewed closely to determine which behaviours were sufficiently frequent and reliable to label manually, including task-specific (e.g. grasping a pellet) and non-specific (e.g. rearing) behaviours. The behaviours were manually labelled using a Jython-based, custom-developed graphical user interface (GUI). For each recording session, videos with different fields of view (with at least one of the performers and one of the observers) were loaded into the GUI, and two experimenters scored behaviours from the same sessions frame by frame. The behaviours used for subsequent neural analyses included nose poke, grasping, eating, grooming, turning (with clockwise and counter-clockwise turning separated) and rearing (Movie S2). In the open field we only quantified wheel-running behaviour, but again discretized clockwise and counterclockwise directions. We also labelled epochs when observer animals moved their limbs or bodies during the observation experiments, allowing us to measure neuronal activity during observer movement.

### Calcium imaging

One photon imaging of intracellular calcium activity was acquired at a rate of 20–25 Hz, with LED power set to 20–30% and a gain of 1; the same image acquisition parameters were maintained for a given set of sessions (4 × 10 min) to allow for comparison of neural activity^28^. Calcium imaging timestamps were synchronized with the behavioural recording system for offline behavioural analyses. Synchronization was done using the nVista DAQ box (Inscopix), which enabled triggering of external hardware (behavioural recording system; Simi) using a TTL system. GCaMP6m-expressing C57BL/6 mice were imaged while performing the pellet-reaching task (2 × 10 min), and again while observing the task (2 × 10 min) while head-fixed. The following day, the same animals were imaged while freely exploring the open field with the running wheel (2 × 10 min), and again while head-fixed, observing a conspecific doing the same (2 × 10 min).

### Image processing

Fluorescence movies were processed using Mosaic Software (v.1.1.2, Inscopix). Raw videos were spatially downsampled by a factor of 4 to reduce file size and processing time; temporal downsampling was not applied. Dropped frames were isolated and interpolated, and the movies were cropped to remove regions lacking cells. For pellet-reaching and open field experiments, performance and observation recordings of the same task were concatenated to generate a single 40 min recording. Motion artefacts were corrected using a single reference image (typically obtained by drawing a border around a large blood vessel or selecting bright neurons) using the Turboreg image registration algorithm within Mosaic software. The movies were further cropped to remove post-registration black borders.

### Fluorescence trace extraction

Motion-corrected, cropped recordings were saved as .tiff files for subsequent signal extraction using the constrained non-negative matrix factorization algorithm for endoscopic recordings (CNMF-E)^66^. CNMF-E was designed to isolate large fluctuations in background fluorescence and facilitate the accurate extraction of cellular signals by simultaneously denoising, deconvolving and demixing one photon calcium imaging data. The CNMF-E framework can be summarized by the following steps: (1) initialize the spatial and temporal components of all neurons without explicit estimation of the background, (2) approximate the background given the activity of all neurons, (3) update spatial and temporal components by subtracting background from the raw image using alternating matrix factorization, (4) delete neurons and merge neurons with high temporal correlations, (5) repeat steps 2–4 (for quantitative detail see ref. ^66^). Similar parameters (gSig = 3, gSiz = 13, mincorr = 0.9) were used across different data sets to extract fluorescence signals. After calcium signal extraction with CNMF-E, fluorescence traces were deconvolved to approximate relative firing rates in each imaging frame using ‘Online Active Set methods for Spike Inference’ (OASIS)^67^. For this, the fluorescence data was modelled using an autoregressive (AR(1)) process due to the fast rising time of calcium. The decay time of the calcium signal (g hyperparameter) was estimated from the autocorrelation, and the optimized g hyperparameter was set to 0. Lastly, a strict threshold of 5 standard deviations from the mean event was used for further calcium event estimation. All subsequent analyses used the inferred calcium events to minimize the effect of decay kinetics of calcium signals.

### Signal-to-noise ratio

A signal-to-noise ratio (SNR) analysis was performed to estimate the quality of the deconvolved output relative to raw traces. Every raw trace value in the interval spanning one second before to seven seconds after a registered calcium event (to accommodate the sharp rise and slow decay of the calcium signal) was considered as signal, and everything outside that range was considered as noise. The SNR was defined as the ratio of the mean of the traces related to calcium events and the standard deviation of the noise. Any cell that failed to exceed or match the SNR minimum value of 3.5 for all sessions was discarded from further analyses.

### Behavioural tuning and shuffling

Calcium event rates were calculated for each cell during each behaviour by dividing the total number of events within a behaviour by the total time spent in that behaviour (in seconds). The calcium event trains were then offset by a random interval between 20 and 60 sec one thousand times, and event rates for each behaviour were re-calculated for each permutation, generating a shuffled distribution. The observed firing rates were z-scored relative to the shuffled distribution, and a cell was considered significantly tuned if its z-scored rate was 2 standard deviations above its shuffled mean during a given behaviour. Only cells meeting this criterion for two of the same type of session were considered stably tuned. During observation sessions, the observers’ body movements were registered in addition to the behaviour of the performer. Cells tuned to the observer’s movement in any session were discarded from the analysis as potentially showing tuning to observed actions.

### Peri-event time histograms

Calcium events were binned in 200 ms windows relative to the onset of a given behaviour, converted into rates and convolved with a Gaussian kernel with a width of 1 bin. Behavioural epochs shorter than 100 ms were excluded from the analysis. For each bin, the mean and the standard error of the mean were calculated over epochs. After averaging over epochs, each cell was normalized to its peak rate, and cells were ordered according to the magnitude of their z-scored rate in the first performing session (P1).

### False-positive estimations

For either brain area, we assessed whether the number of stably tuned neurons across different conditions (performance, observation and matched) was statistically different from chance (i.e. false positive) rates. The false-positive rate was estimated empirically by swapping behavioural labels between two sessions of the same kind (e.g. O1 and O2) and re-computing calcium rates for each behaviour, thus determining the “false” proportion of stably tuned cells across all animals. The significance of the difference between the distributions of true and false positive proportions was determined with the Mann-Whitney U test.

### Correlation matrices

To assess the predictability of representations across different session types, data from all animals within a region were pooled and significantly tuned cells for each behaviour (e.g. rearing) in each session (e.g. P1) were selected. A given z-scored calcium rate series (e.g. all rearing cells in P1) was then correlated with the series of z-scored rates of all the behaviours in all the other sessions (e.g. all grooming cells in O1).

### Cell registration

To identify discrete states in neuronal population activity using dimensionality reduction (Uniform Manifold Approximation and Projection, UMAP^29^), the stability and identity of cells across all sessions were first confirmed using methods recently published by Sheintuch *et al*.^68^, which uses a probabilistic approach to register the spatial location of cells across sessions. After extraction of spatial components of the imaged data for each recording, spatial footprints were loaded into a graphical user interface (GUI) provided by Sheintuch *et al*. (2017) for further alignment and characterization of the similarity measure. For this analysis, a pixel value of 2.3 µm, maximal distance of 15 µm (due to sparsity) and Psame threshold of 0.95 (to be conservative) were used.

### Decoding

The event data were first smoothed with a Gaussian kernel (10 bin width). Reference population vectors were created for background neural activity and forceps events. The events from the held-out data were classified according to which population vector was closest in Euclidean space, and compared with the real forceps events, yielding the fraction of time points correctly labelled. This was repeated for each fold of the three-fold cross-validation. To create an approximate distribution of what one would expect by chance, we shifted the activity of each neuron independently 10000 times by a random interval between 50 s and 5 min and repeated the same procedure as with the original data.

### Dimensionality reduction

The fast, non-linear dimensionality reduction algorithm, UMAP, was applied to visualize the high-dimensional neural state space using a lower-dimensional manifold while preserving high-dimensional local and global structures. To do this, cells were first registered across a total of 60 minutes of combined pellet-reaching and open field recordings (described in “Cell registration”) to ensure similarity. The calcium event trains were convolved using a Gaussian kernel with a width of 2 bins. Neural data were downsampled to every 2 bins, then further downsampled by keeping only the time points when >10% of the population for performing sessions and >5% for observing sessions had non-zero convolved events. Dimensionality reduction with UMAP was performed assuming a Manhattan distance metric, and the parameters (n_neighbors = 5, min_distance = 0.5, spread = 1.0) were kept the same for all neural data sets.

### Dunn index

The compactness of the behavioural clusters (i.e., cluster of time points corresponding to the same hand-labelled behaviour) in the dimensionality-reduced representation was assessed using the Dunn Index (DI)^69^. To this end, the centroids were first calculated for every behavioural cluster. Distances between each point within a behavioural cluster and the cluster’s centroid (intra-cluster distances) and the distances between centroids of different clusters (inter-cluster distances) were measured. The DI was then calculated as the ratio between the minimum inter-cluster distance and the maximum intra-cluster distance (as defined above). The DI provides a measure of overall clustering quality, i.e., a high Dunn index corresponds to tight clustering in the data.

### Generalized linear model

For performed behaviours, the neural calcium event data from performing sessions was fitted with generalized linear models (GLMs) to determine whether a given performed behaviour explained the calcium events better than the neurons’ mean calcium events rate. The calcium event data were then fitted with a Bernoulli GLM^70^ assuming the neurons were independent. Each GLM contained a parameter corresponding to a hand-labelled behaviour (nose poke, pellet grasping, eating, grooming, turning CW, turning CCW, rearing, running CW or running CCW) as well as a constant term. The log-likelihood of the data given each of the models was maximized across 10 folds of the data. Calcium events recorded from each neuron were also fitted with a Bernoulli GLM (which we call the null model) with only the constant term, which corresponded to the neuron’s mean calcium event rate. The out-of-sample log-likelihood was calculated for each fitted GLM. The cross-validated pseudo-*R*^2^ ^36^ was then calculated as the difference between the out-of-sample null model log-likelihood, which was from the GLM with only the constant term, and the out-of-sample model log-likelihood, which was obtained from the GLM with a parameter attached to a hand-labelled behaviour, normalized over the out-of-sample null model log-likelihood and averaged over 10 folds of the data.

For observed behaviours, the neural calcium event data from observing sessions were also fitted with Bernoulli GLMs to determine whether a given observed behaviour could account for the calcium events beyond what can be explained with the observer’s own behaviour (i.e., body movement). The cross-validated pseudo-*R*^2^ was calculated as with performed behaviours, but with the out-of-sample model log-likelihood obtained from the GLM with parameters attached to an observed behaviour and to body movement, plus the out-of-sample null model log-likelihood from the GLM with a parameter attached to only the body movement.

### Spatial clustering of behaviourally selective neurons

To calculate the spatial distribution of significantly tuned neurons, each neuron’s centroid location was first identified using TrakEM2 software^71^. To do this, neurons identified as responsive to any given behaviour were stacked together in ImageJ, and image stacks for each behaviour were averaged to obtain a single image with the physical locations of tuned neurons. These images were subsequently loaded into TrakEM2 and the position of each cell in each image was manually traced as a circle. The XY location of each circle was calculated to obtain the position of each cell in each animal. Next, Euclidean distances between stably tuned cells in the imaging field for each mouse were calculated. To evaluate spatial clustering of cells based on their behavioural correlates, the Dunn Index (DI; see Dimensionality reduction: Dunn index) of each animal’s recorded dataset was compared against the distribution of DIs generated from shuffled data. A behavioural cluster was defined as the cluster of cells that was stably tuned to a given behaviour. The shuffled distribution of DIs was obtained by randomly permuting cell IDs one thousand times and recalculating the DI for each permutation.

### Anatomical verification of imaging locations

For perfusions, animals were anaesthetized deeply using isoflurane (5%) and subsequently injected with sodium pentobarbital (200 mg/kg; intraperitoneal injection) and transcardially perfused using ~25 ml saline followed by ~50 ml of 4% paraformaldehyde (PFA). Each mouse was decapitated and the brain was removed carefully from the skull. Brains were kept in 4% PFA at 4 °C overnight, then transferred to 2% dimethyl sulfoxide (DMSO: VWR, Radnor, PA) solution for cryoprotection for 1–2 days. The brains were cut in coronal sections in 3 series of 40 µm on a freezing sliding microtome (HM-430 Thermo Scientific, Waltham, MA). The first series was mounted directly onto the superfrost slides (Thermo Scientific) to perform Nissl-staining for delineation purposes. The remaining series of sections were collected in vials containing 2% DMSO and 20% glycerol in phosphate buffer (PB) and stored at −20 °C until further usage.

For immunohistochemical staining, the second series of sections was used to visualize GCaMP6m viral expression. The brain sections were first rinsed 3 × 5 min in PBS on a shaker, incubated in blocking buffer (PBS plus 0.3% Triton, 2 × 10 min), followed by incubation in primary antibody solution (rabbit anti-GFP, 1:1000, ThermoFisher Scientific, A-11122, in PBS and 0.3% Triton) overnight at 4 °C. Sections were further washed in PBS containing 0.3% Triton and 3% bovine serum albumin (BSA; Sigma Aldrich) for 2 × 5 min at room temperature (RT), and subsequently incubated in secondary antibody solution (AlexaFluor 488-tagged goat anti-rabbit Ab, 1:1000, ThermoFisher Scientific, A-11008) for 1 h at RT. Sections were washed 2 × 10 min in PBS and mounted on gelatin-coated polysine microscope slides and dried in the dark overnight. Next, sections were treated with Hoechst solution (1:5000; Sigma Aldrich) for 5 min in the dark and immediately rinsed with PBS. Slides were air-dried overnight in the dark at RT and cover-slipped using entellan-toluene solution (Merck Chemicals) the following day.

For anatomical delineation of recording locations, all brain sections were digitized using an automated scanner for fluorescence and brightfield images at the appropriate illumination wavelengths (Zeiss Axio Scan.Z1, Jena, Germany). Corresponding Nissl stained sections were used to delineate PPC, M2 and neighbouring cortical regions in each animal in accordance with Hovde *et al*. (2018), the borders of which were copied onto the GFP-stained images in Adobe Illustrator CC 2017. Bregma coordinates were estimated in correspondence with Paxinos & Franklin^72^.

### Ethics declarations

Experiments were performed in accordance with the Norwegian Animal Welfare Act and the European Convention for the Protection of Vertebrate Animals used for Experimental and Other Scientific Purposes.

## Supplementary information

Supplementary material; Figures S1–S7 and Table S1.

Movie S1.

Movies S2.

## Data Availability

The datasets generated during the current study are available from the corresponding authors upon reasonable request.

## Code availability

Code for reproducing the analyses in this article is available from the corresponding authors upon reasonable request.
