# Supplementary material for: Action representation in the mouse parieto-frontal network
Source: Sci Rep. 2020 Mar 27;10:5559. doi: 10.1038/s41598-020-62089-6 (PMC7101420; doi:10.1038/s41598-020-62089-6)
Supplement: Supplementary file 1 — Supplementary material; Figures S1–S7 and Table S1. [file 41598_2020_62089_MOESM1_ESM.pdf]

Supplementary Material:

Action representation in the mouse parieto-frontal network

Tuce Tombaz†*, Benjamin A. Dunn*, Karoline Hovde§, Ryan J. Cubero§, Bartul Mimica§, Pranav Mamidanna, Yasser Roudi, Jonathan R. Whitlock†

* These authors contributed equally to the work

§ These authors contributed equally to the work

† Correspondence

Supplementary Figures 1-7

Supplementary Table 1

Supplementary Figure legends

Supplementary Movie legends

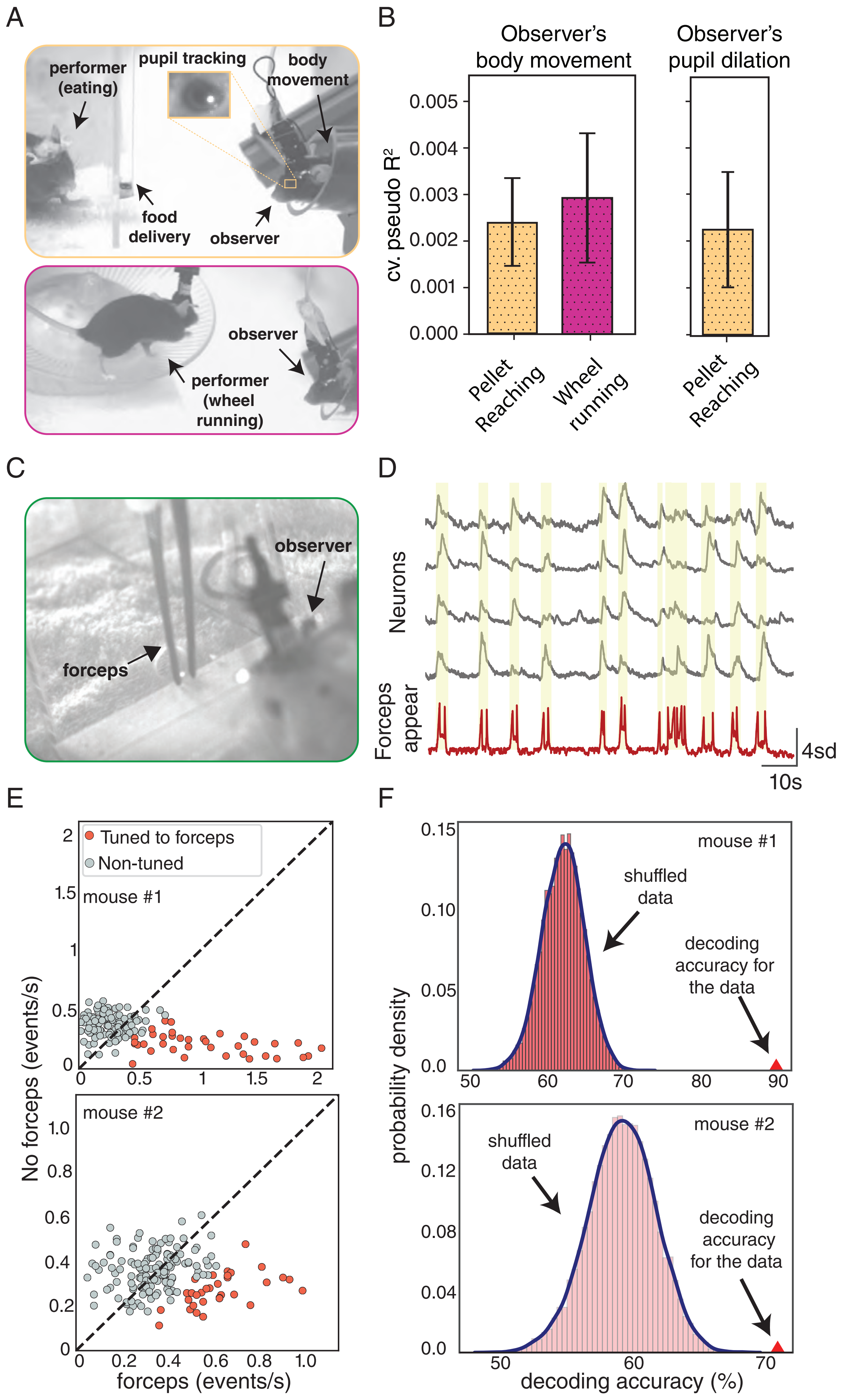

Supplementary Fig. S1

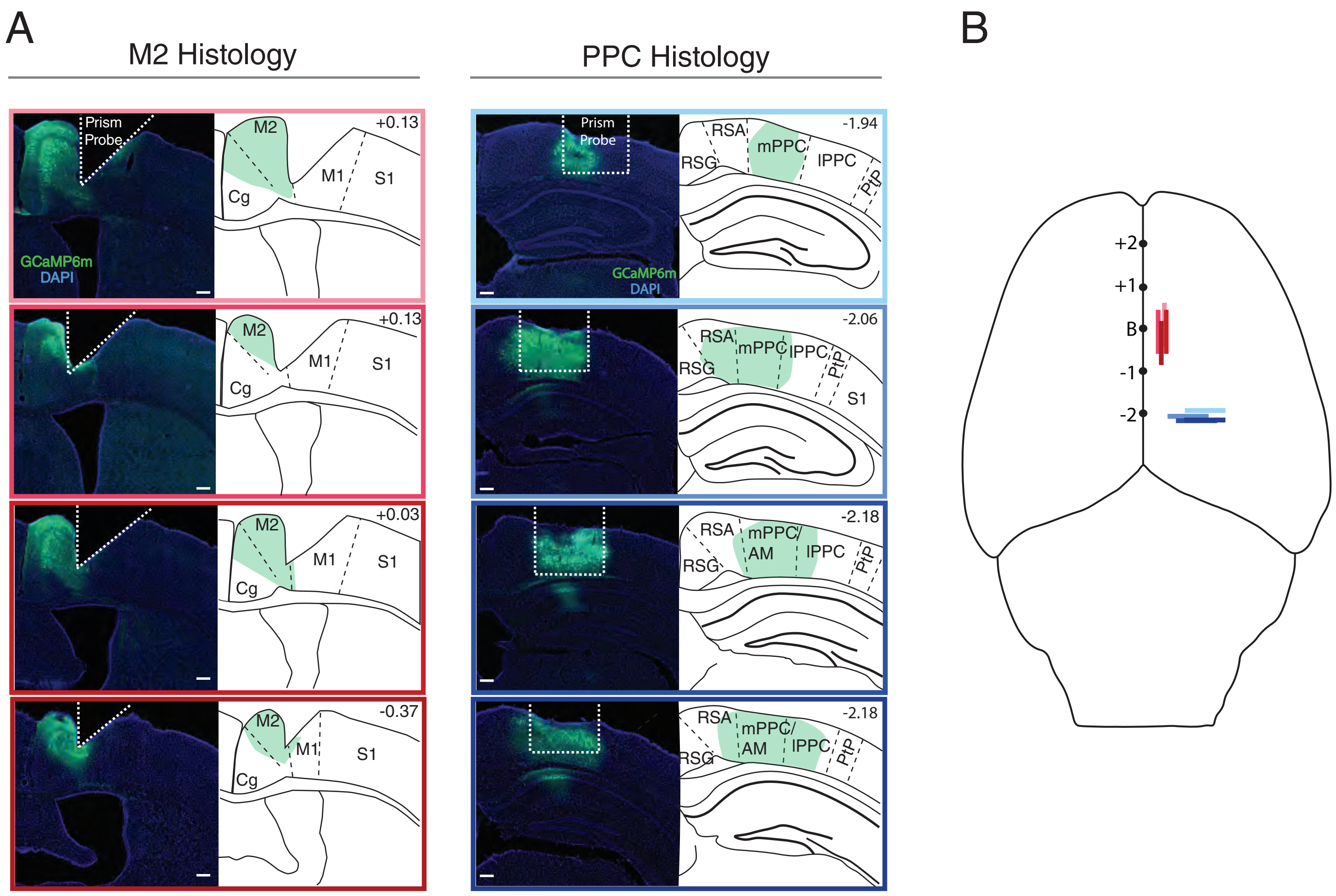

Supplementary Fig. S2

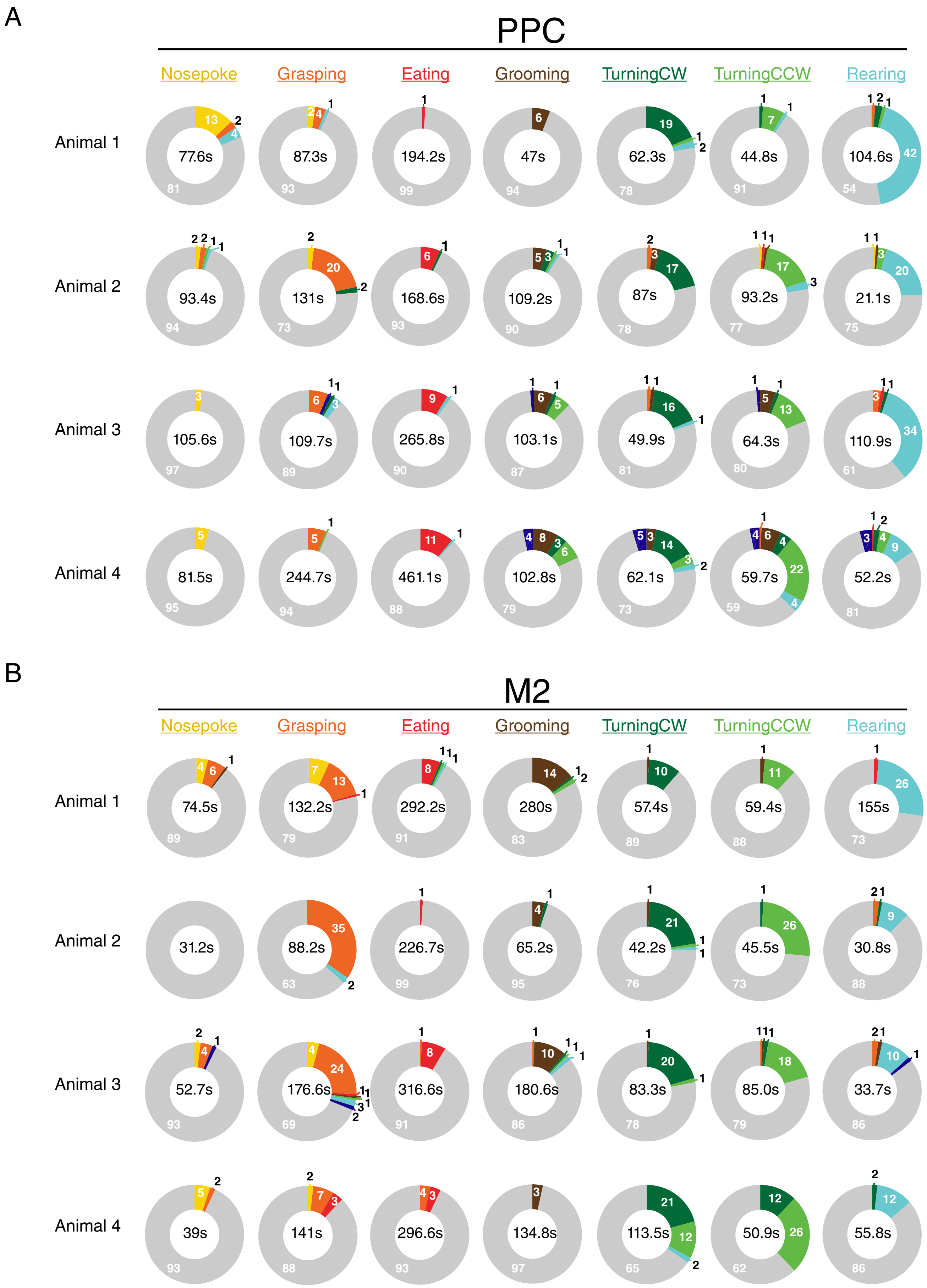

Supplementary Fig. S3

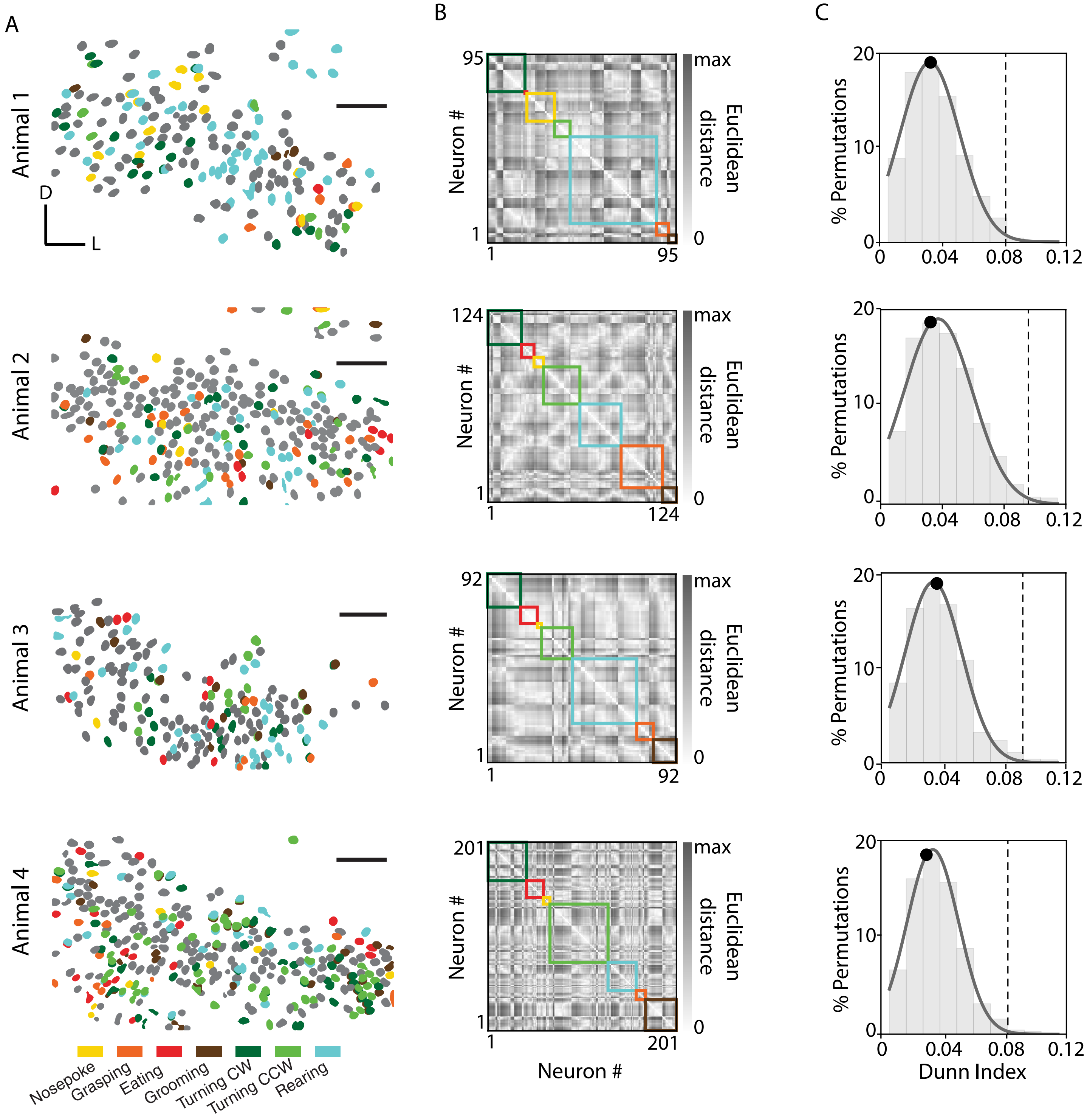

Supplementary Fig. S4

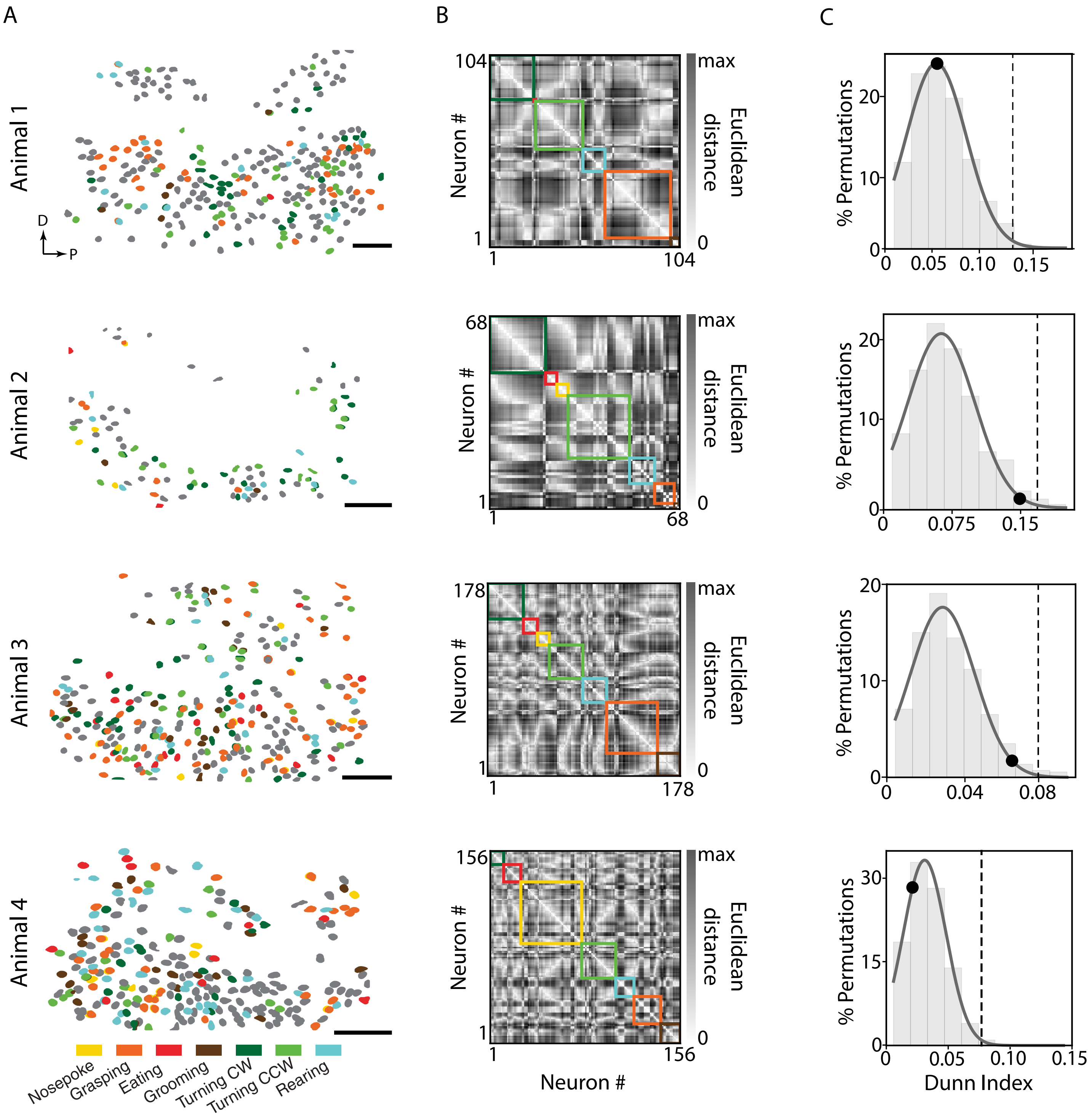

Supplementary Fig. S5

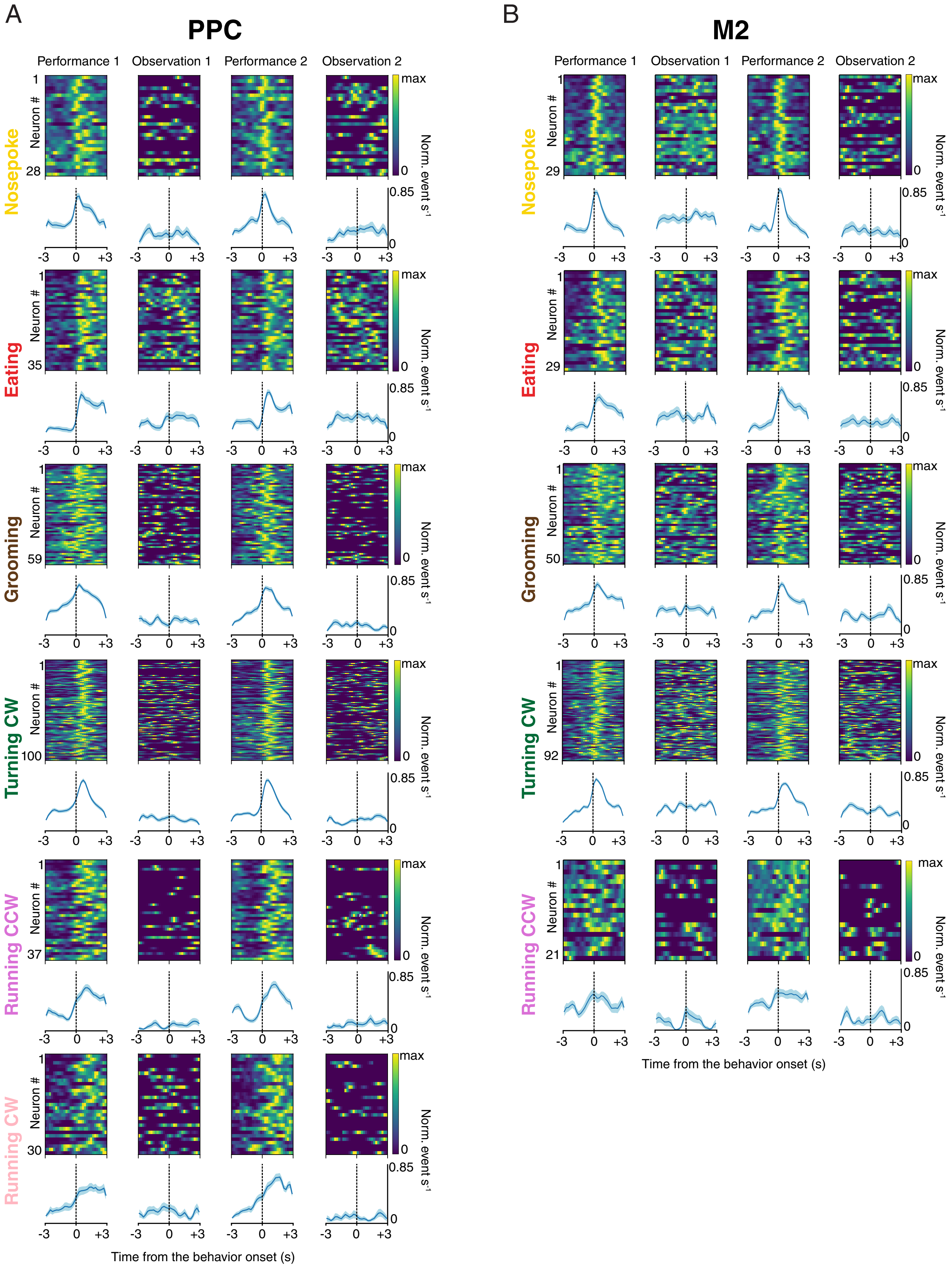

Supplementary Fig. S6

A

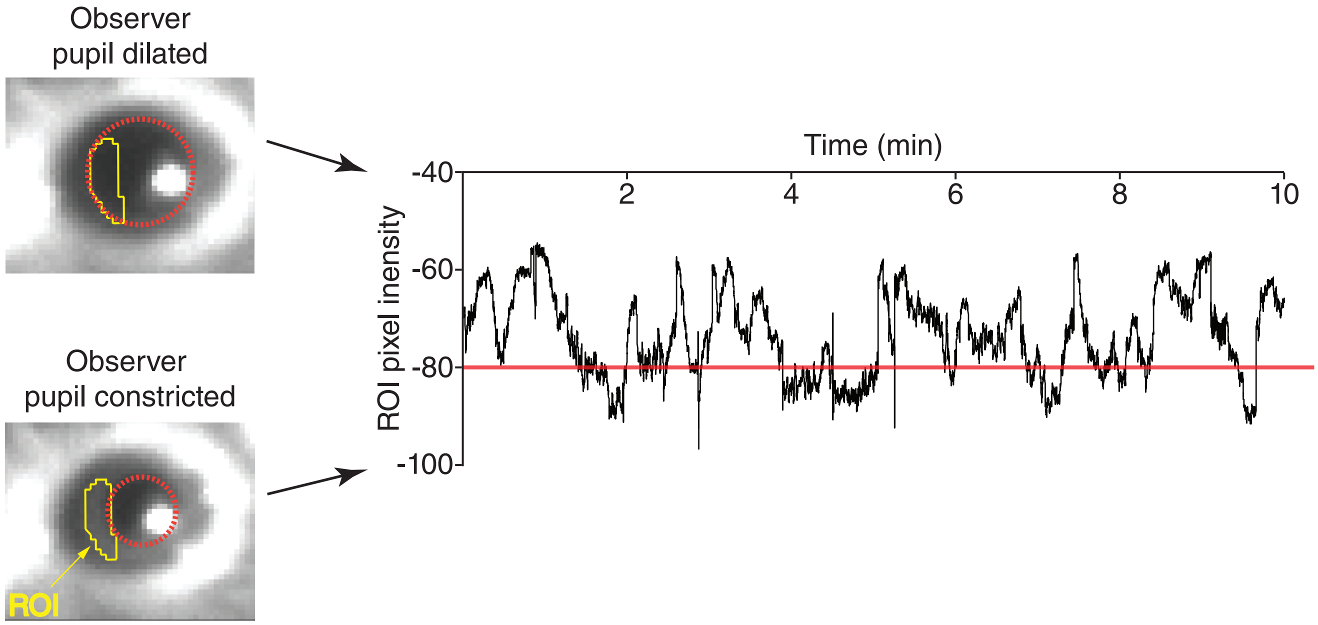

B

M2

		# cells	performance	observation	matched
Whole Session	Animal 1	264	98 (37.1%)	4 (1.5%)	1 (0.4%)
	Animal 2	99	58 (58.6%)	1 (1.0%)	0
	Animal 3	249	151 (60.6%)	4 (1.6%)	2 (0.8%)
	Total	612	307 (50.2%)	9 (1.5%)	3 (0.5%)

		# cells	performance	observation	matched
Excluding pupil constriction	Animal 1	264	98 (37.1%)	4 (1.5%)	0
	Animal 2	99	58 (58.6%)	0	0
	Animal 3	249	151 (60.6%)	4 (1.6%)	1 (0.4%)
	Total	612	307 (50.2%)	8 (1.3%)	1 (0.2%)

Supplementary Fig. S7

Table S1. Summary of performance and observation tuning in PPC and M2

PPC

		# cells	performance	observation	matched
Pellet Reaching	Animal 1	177	85 (48%)	1 (0.6%)	0
	Animal 2	268	111 (41.4%)	4 (1.5%)	0
	Animal 3	179	80 (44.7%)	3 (1.7%)	1 (0.6%)
	Animal 4	297	154 (51.9%)	7 (2.4%)	2 (0.7%)
	Total	921	430 (46.7%)	15 (1.6%)	3 (0.3%)

		# cells	performance	observation	matched
Wheel-Running	Animal 1	149	20 (25.5%)	0	0
	Animal 2	337	37 (10.9%)	1 (0.3%)	0
	Animal 3	238	10 (4.5%)	2 (0.8%)	0
	Total	724	67 (9.3%)	3 (0.4%)	0

M2

		# cells	performance	observation	matched
Pellet Reaching	Animal 1	264	98 (37.1%)	4 (1.5%)	1 (0.4%)
	Animal 2	99	58 (58.6%)	1(1.0%)	0
	Animal 3	249	151 (60.6%)	4 (1.6%)	2 (0.8%)
	Animal 4	240	132 (55%)	4 (1.7%)	0
	Total	852	439 (51.5%)	13 (1.5%)	3 (0.4%)

		# cells	performance	observation	matched
Wheel-Running	Animal 1	216	21 (9.7%)	1 (0.5%)	0
	Total	216	21 (9.7%)	1 (0.5%)	0

Supplementary Table S1

Supplementary Figure legends

Fig. S1. (*A*) Snapshots from pellet reaching and open field tasks show the placement of the animals during performance and observation conditions. The pupils of a subset of observers were recorded during the pellet reaching task (top). (*B*) Bar plots showing the cross-validated pseudo-R^2 of a GLM predicting the observers' body movement (left) or pupil dilation (right) as a function of whether the performers were pellet reaching (comprised of nose poke, grasping and eating, *yellow bars*) or wheel running (*pink bar*). All mice in the study were pooled for the analysis; error bars indicate ± SEM. (*C*) Control experiments where PPC activity was imaged in two observing mice while forceps repeatedly delivered and removed food pellets at the reaching hole of an empty pellet-reaching box. (*D*) Black traces show four examples of visually responsive PPC neurons stimulated by the appearance of the forceps. The red trace (below) denotes when the forceps were present in front of the mice; yellow bars indicate the time intervals when the forceps were present. (*E*) Comparisons of calcium event rates for single neurons between "forceps-present" and "no-forceps" conditions for both animals from *C*. (*F*) Population vector decoding of forceps appearance for the same mice as in *C*. The probability density functions show the shuffled data (fitted with a Gaussian curve, *blue*), and the red triangle denotes the decoding accuracy for each mouse.

Fig. S2. (*A*) (*Left*) Histological sections (40µm) showing GCaMP6m expression in M2, with prism probe locations depicted by the white dashed line. (*Right*) Same, for animals in PPC. In both areas, schematics of the tissue were drawn to show the extent of GCaMP6 expression in green. Anatomical boundaries for PPC, M2 and surrounding regions were established using lamination and cytoarchitectural profiles in adjacent, Nissl-stained sections. Scale bar denotes 200 µm. (*B*) Dorsal view of estimated recording planes in M2 (red rectangles) and PPC (blue) in all 8 animals. Bregma ("B") is indicated on the midline, and black dots indicate 1 mm.

Fig. S3. Subsets of cells in PPC and M2 were stably tuned to multiple behaviours. (*A*) Colour-coded pie charts show the proportion of PPC neurons significantly tuned to each behaviour in the pellet reaching task, with the percentage of cells in each category written around the ring periphery, and the total time in each behaviour (summed across both performance sessions) shown in the centre. To display the relative proportions graphically, cells tuned to multiple behaviours (e.g. “Nose poke” and “Grasping”) appear in more than one pie chart. Cells stably tuned to three or more behaviours are denoted by dark blue, while cells not tuned to the behaviour of interest are shaded in grey. (*B*) Same as in *A*, but for M2.

Fig. S4. Behaviourally tuned neurons in PPC did not cluster anatomically. (*A*) Cell maps for each animal, colour-coded by their behavioural correlates (legend at bottom). Scale bars = 100µm. (*B*) Matrices showing pairwise Euclidean distances between neurons grouped by their tuning preferences (coloured boxes); shortest distances are shown in white and longer distances are darker. Functional-anatomical clustering would produce lighter shading within-behaviour and darker colours outside. (*C*) The quality of clustering by behaviour was quantified using the Dunn index (Methods), which assessed Euclidean distances between cells with similar vs. different behavioural classifications. The distribution of actual intra- vs. inter-cluster distances was compared against a shuffled distribution in which cell identities were permuted, which indicated below-chance levels of clustering in each animal. Dashed lines indicate the 99th percentile of the shuffled distribution; black circles denote the Dunn index value.

Fig. S5. Behaviourally tuned neurons in M2 were not clustered anatomically. (*A*) Cell maps for each animal, colour-coded by their behavioural correlates (legend at bottom). Scale bars = 100µm. (*B*) Same matrices as for PPC cells in Figure S4, showing pairwise Euclidean

distances between neurons grouped by tuning preferences. (*C*) The quality of clustering by behaviour was quantified using the Dunn index (Methods), as with PPC neurons in the previous Supplementary figure; none of the animals showed neural clustering exceeding the 99th percentile of the shuffled distribution (dashed lines); black circles denote the observed Dunn index value.

Fig. S6. Additional behavioural conditions in relation to Figure 4 comparing PPC and M2 ensemble activation during performance and observation sessions. (*A*) As with Figure 4, PPC cells responded during performed, but not observed actions. (*B*) Same as *A*, but for cells recorded in M2; insufficient data were collected to test for stable tuning for Running CW for recordings in M2, so that condition was omitted. Note that the behaviours here are included in the cross-correlation matrices for performance and observation sessions in Figure 4 *C* and *D*.

Fig. S7. Arousal state did not influence neural responses to observed actions. (*A*) Pupil size was measured as a proxy for arousal state during observation of the pellet-reaching task in three mice with prisms in M2. (*Left*) A region of interest (ROI) was drawn over a close-up video of the eye using ImageJ software, and pupil size (red circle) was reported via pixel intensity inside the ROI (*Right*). For each mouse, a threshold was determined to capture epochs when the pupil was constricting to its smallest size (red line in graph), typically when animals were quiescent and motionless. (*B*) The number of cells with stable correlates for observed behaviour was below the false positive rate regardless of whether epochs with small pupil diameter were included in the analysis.

Supplementary Movies

Movie S1. (left panel) The momentary state of neural population activity is indicated by the blue cursor, while the dimensionally-reduced manifold of population activity for the entire session is shown as grey dots. Darker areas correspond to denser regions in the reduced space. Note that the cursor (i.e. the state of population activity) occupies a stable location when the animal performs clockwise and counter-clockwise running, but that it moves unpredictably over the manifold when the same animal observes a cohort running on the wheel. (right panel) Corresponding in-session videos of wheel-running epochs from performance and observation sessions. For display purposes, calcium events were convolved with a Gaussian kernel with a width of 5 bins before downsampling and manifold learning using UMAP (Methods).

Movie S2. Video showing a side-view of a mouse performing the pellet reaching task. Each behaviour included in the neural data analyses is demonstrated in the video.
